# Parallel Logic Operations in Electrically Tunable Two-Dimensional Homojunctions

**DOI:** 10.1021/acs.nanolett.4c04337

**Published:** 2024-10-30

**Authors:** Yuliang Chen, Zhong Wang, Chongwen Zou, Stuart S. P. Parkin

**Affiliations:** † Max Planck Institute of Microstructure Physics, 06120 Halle, Germany; ‡ National Synchrotron Radiation Laboratory, School of Nuclear Science and Technology, University of Science and Technology of China, 230029 Hefei, China

**Keywords:** 2D semiconductor, logic device, p−n junction, encryption, parallel logic operation

## Abstract

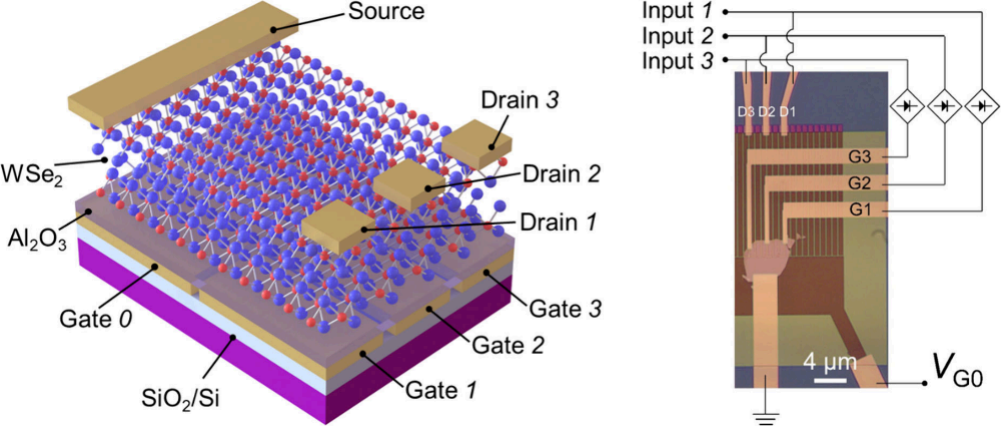

Two-dimensional materials show great potential for future electronics beyond silicon materials. Here, we report an exotic multiple-port device based on multiple electrically tunable planar p–n homojunctions formed in a two-dimensional (2D) ambipolar semiconductor, tungsten diselenide (WSe_2_). In this device, we prepare multiple gates consisting of a global gate and several local gates, by which electrostatically induced holes and electrons are simultaneously accumulated in a WSe_2_ channel, and furthermore, at the boundaries, p–n junctions are formed as directly visualized by Kelvin probe force microscopy. Therefore, in addition to the gate voltages in our device, the drain/source bias can also be used to switch the 2D WSe_2_ channel on/off due to the rectification effect of the formed p–n junctions. More importantly, when the voltage on the global gate electrode is altered, all p–n junctions are affected, which makes it possible to perform parallel logic operations.

Silicon-based metal–oxide–semiconductor field-effect transistors (MOSFETs) are the basic electronic components of today’s logic circuits. Continually shrinking their size over the past 4 decades has led to dramatically increased performance.^1,2^ However, the further shrinkage in the size of these silicon-based transistors has met their physical limits. Hence, the development of electronic chips based on novel materials and technologies has become of high importance.^3−6^ 2D materials, stacked layer by layer via van der Waals forces, have attracted considerable attention owing to their remarkable physical properties^7^ and are considered to be one of the most promising candidates for next-generation electronics.^8^ In particular, transition metal dichalcogenides (TMDs) that display semiconducting properties are an ideal platform for 2D nanoelectronics.^9,10^ Nanodevices based on TMD homo- and heterojunctions, which have been fabricated by transfer techniques^11−13^ and direct growth,^14,15^ are prospective candidates for random access memories, logic elements, and neuromorphic devices and circuits.^16−27^ Electrically tunable homojunctions (ETH) with ambipolar TMD channels show highly interesting field-effect characteristics,^28−31^ in which holes and electrons are independently electrostatically doped into a 2D channel by two gates, resulting in a p–n homojunction at the boundary. In ETH devices, not only the gates but also the drain/source bias are knobs to switch the 2D channel on/off due to the rectification effect of the p–n junction, and these novel physical principles at the component level allow for advanced logic circuits. To this end, various logic gates have been built by cascading multiple ETH devices.^32,33^ Although ETH devices can, in principle, carry out similar functions as MOSFET-based logic circuits, the integration of ETH devices faces challenges in complicated cascading designs and preconfigurations owing to limited ports per device because usually only one p–n junction is formed in a single ETH device.^20,23,25,32−37^

Here, we report a novel multiple-port ETH device for parallel logic operations using WSe_2_, an ambipolar TMD, as the channel material. As distinct from previous ETH devices with two input ports due to one p–n junction,^18−20,23,25,33,35,36^ our multiple-port devices possess several p–n junctions in a single ETH device resulting from a unique multiple-gate design consisting of a global gate and multiple local gates. These p–n junctions can be dynamically formed and/or erased by independently tuning each gate voltage, which has been directly visualized by Kelvin probe force microscopy (KPFM). Furthermore, when the voltage on the global gate electrode is altered, all p–n junctions are affected. Accordingly, parallel logic operations are anticipated using our unique multiple-port ETH device. Indeed, in experiments, we demonstrate that multiple inputs can be exerted simultaneously in logic operations in one multiple-port ETH device and can be extended to practical applications such as information encryption. In addition, symmetric and asymmetric encryptions based on logic operations are implemented in a confidential communication route integrated with two multiple-port ETH devices. Our studies have not only realized the direct observation of electrically tunable 2D p–n junctions but also demonstrated a practical and scalable 2D unit for multiple-terminal logic computation, which shows unique advantages for future integration circuit fabrication.

## Structure and KPFM Characterization

**Figure 1:**
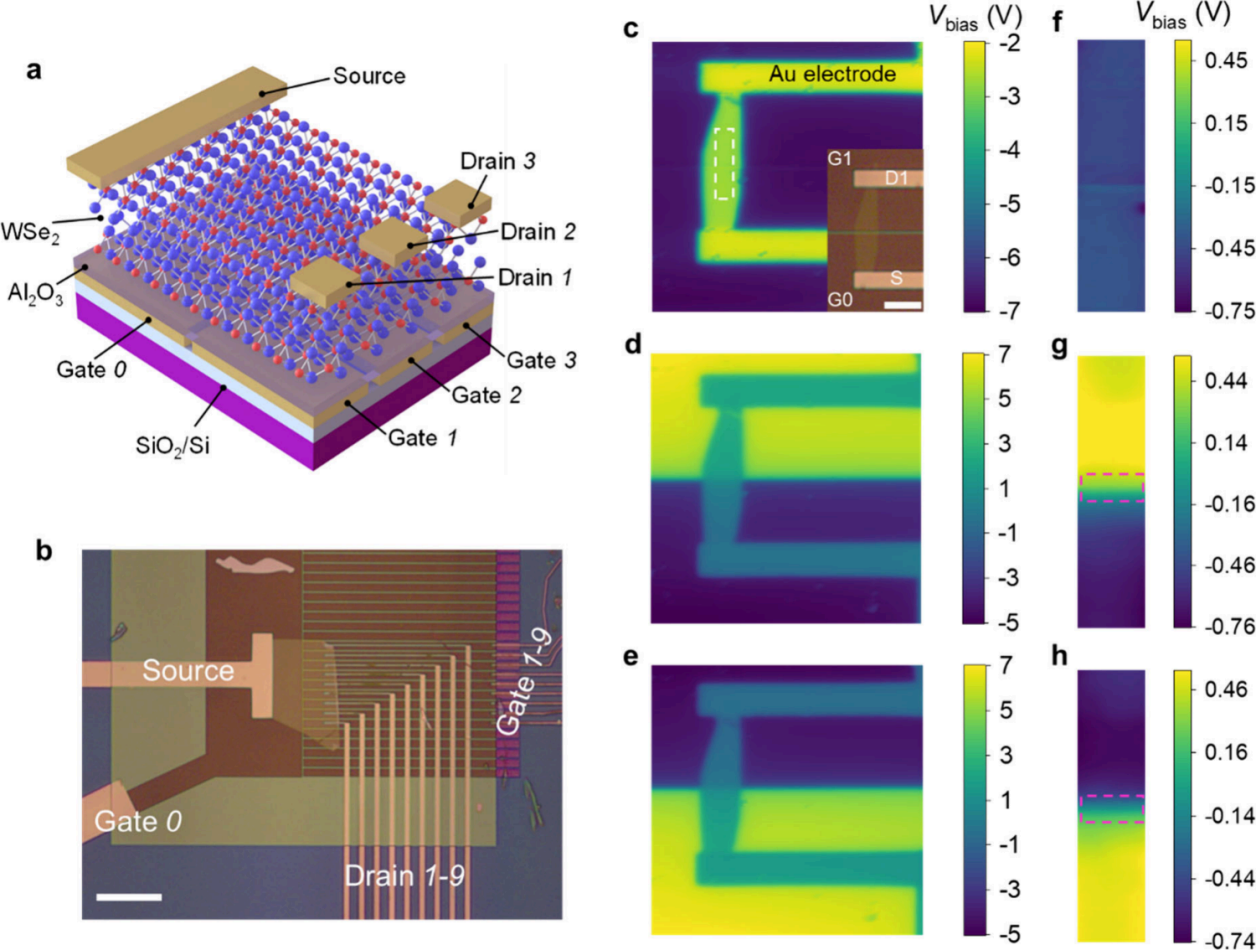
Structure of the ETH device and KPFM results. (a) Schematic illustration of a multiple-port ETH device with a WSe_2_ bilayer channel. Red and blue balls represent W and Se, respectively. (b) Optical image of a 10-gate ETH device. Scale bar: 10 μm. (c–e) KPFM results from device 1 (shown in the optical micrograph in the inset of panel c (scale bar: 4 μm)) for different gate voltages. *V*_G0_ = *V*_G1_ = −8 V (c), *V*_G0_ = −8 V and *V*_G1_ = 8 V (d), *V*_G0_ = 8 *V* and *V*_G1_ = −8 V (e). (f–h) Extracted data from (c–e) as indicated by the white dashed box of area 1.25 μm × 5 μm in (c) are replotted in (f–h), respectively. We use *V*_bias_ of the Au electrodes as a reference for processing the experimental data.The pink dashed boxes in (g, h) mark the p–n junctions.

A schematic diagram of the multiple-port WSe_2_ ETH device is shown in Figure 1a. Multiple split gate electrodes are designed with one global gate (G0) and several local gates (G1, G2, and G3) in parallel with one another (see details in Figure S1a; note that previous ETH devices only have two gates,^28−30^ e.g., Figure S1c,d and the inset of Figure 1c). The separation between the global gate and the local gates is ∼230 nm (see scanning electron microscope (SEM) image in Figure S1b). An Al_2_O_3_ dielectric layer is deposited to cap all gate electrodes, and then a few-layered WSe_2_ flake as the channel is transferred on top of the Al_2_O_3_ layer. Finally, one source electrode (S) and multiple drain electrodes (D1, D2, and D3) are fabricated on the WSe_2_ flake at the positions defined by the corresponding bottom gates. Figure 1b shows an optical image of a 10-gate device formed with a WSe_2_ bilayer (∼1.4 nm) flake.

When a voltage is applied between the gates and the source, electrostatic doping takes place. We visualize the accumulated holes or electrons in the WSe_2_ channel by KPFM, which examines the local variation of the surface potential with high resolution. The potential difference between the KPFM tip and the sample causes the tip to mechanically oscillate which is compensated by applying a DC bias voltage (*V*_bias_) whose magnitude is related to the work function of the sample.^38−40^ When electrons accumulate in the WSe_2_ channel, empty band states are occupied, which thereby reduces the work function and otherwise increases when holes accumulate.

To facilitate the KPFM tests, we measured the formation of a single junction between one local gate and the global gate. Hence, we used a 2-gate WSe_2_ bilayer device as previous 2-port ETH devices^28−31^ with one global gate G0 and one local gate G1 (device 1, inset of Figure 1c and Figure S1c,d). During the tests, the S and D1 terminals on the WSe_2_ channel were shorted and grounded. We first consider symmetric configurations; i.e., the same gate voltages are applied to G0 and G1. In Figure 1c,f, when −8 V was applied to G0 and G1, the results showed that no junction was produced, but, rather, significant hole accumulation occurred in the entire WSe_2_ channel because *V*_bias_ was lowered by 0.5 V compared to that of the Au electrodes (Figure S2p). The work function of intrinsic WSe_2_ is 4.3 eV, and Au has a 5.1 eV work function.^41^ If there is no hole doping, WSe_2_ should have a higher *V*_bias_ than that of Au, which was also confirmed via a test at *V*_G0_ = *V*_G1_ = 0 V (Figure S2d,h). Additional KPFM results from symmetric configurations of G0 and G1 show that not only the doped charge types can be switched between hole and electron by switching the polarities of gating voltages but also the amount of charges accommodated in the WSe_2_ channel can be successively adjusted by tuning the magnitude of the gating voltages (Figures S2 and S3). Notably, the offset of *V*_bias_ between *V*_G0_ = *V*_G1_ = 8 V and *V*_G0_ = *V*_G1_ = −8 V is up to 1.2 eV, which is consistent with the 1.3 eV bandgap of WSe_2_ as in previous reports,^41,42^ which means that electrostatic doping can shift the Fermi level (*E*_F_) into the valence band or the conduction band across the whole bandgap. Accordingly, the KPFM results strongly suggest that our device is anticipated to become an ambipolar FET by means of symmetric configurations of the gate voltages.^9,43^

Furthermore, different voltages, i.e., asymmetric configurations, can also be applied to G1 and G0. KPFM demonstrated that different polarities of *V*_G0_ and *V*_G1_ allow for holes and electrons to be simultaneously accumulated in distinct WSe_2_ segments. In Figure 1d,g, where *V*_G1_ = −*V*_G0_ = 8 V, electrons (holes) were accumulated in the top (bottom) half segment because of the positive (negative) voltage on G1 (G0). Swapping the polarities of the gate voltages results in opposite charge accumulations (Figure 1e,h). Since holes and electrons are accumulated in different portions of the WSe_2_ channel, a p–n homojunction is formed at their boundary. A depletion region ∼600 nm in width was visualized in the form of a successive evolution of *V*_bias_ along the WSe_2_ channel (pink dashed boxes in Figure 1g,h and Figure S4). We also tested another device (device 2), showing KPFM results similar to those of device 1 (Figure S5). Note that here we used one local gate with the global gate G0 to produce one junction, but if more local gates are used, more junctions can be produced, for example, a 10-gate device can form 9 junctions as shown in Figure 1b. However, subjected to the limited capacity of our KPFM device, we did not measure this device.

## Electrical Measurements and Physical Principles for Logic Operations

**Figure 2:**
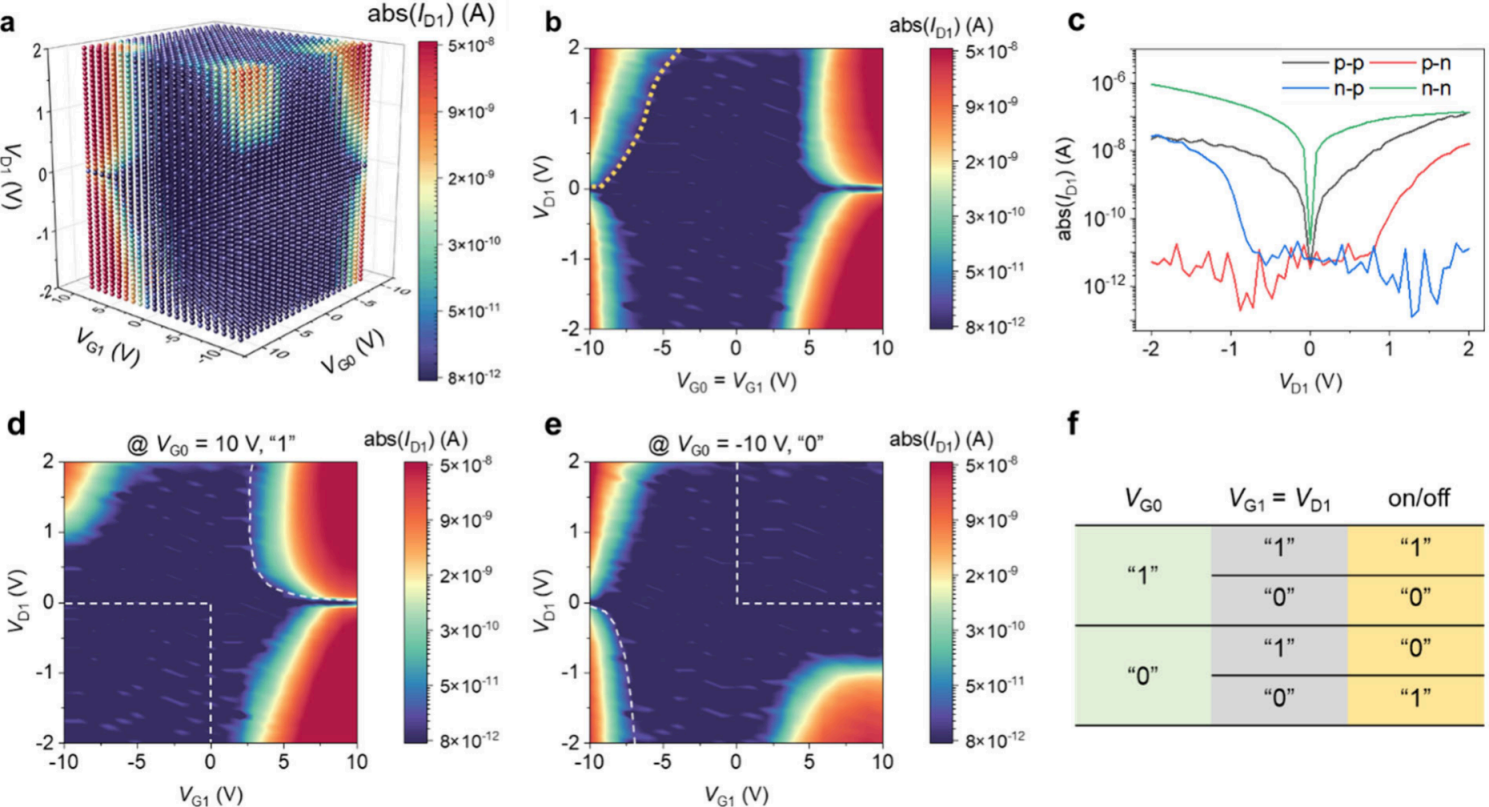
Electrical measurements and logic principles for ETH devices. (a) A set of electrical results from device 1. (b) Diagonal plane of (a) (*V*_G0_ = *V*_G1_). (c) Four vertical edges of (a), indicating reconfigurable rectifications. “n” and “p” indicate electron and hole doping in the two regions of the WSe_2_ channel, respectively. (d–f) Extracting the logic rules. Front surface (d) of (a) (*V*_G0_ = 10 V in logic state “1”) and back surface (e) of (a) (*V*_G0_ = −10 V in logic state “0”) suggest that the logic states carried by a pair of drain/gate can be dynamically converted by *V*_G0_. The logic rules are concluded in (f). The dashed lines in the first and third quadrants of (d, e) are for highlighting the available regions for logic rules. The yellow dots in (b) give an example for defining the thresholds, which can also work in (d, e).

The KPFM results show that the p–n homojunction in the ETH device can be dynamically formed/erased if the gate voltages are switched between the symmetric and asymmetric configurations. In the following, we performed electrical measurements on device 1 based on its KPFM results. In the symmetric (asymmetric) configurations of gate voltages, we predict that the conductive states of the channel are independent (dependent) of the polarity of readout bias applied between the source and drain electrodes due to only one (two) type(s) of charges that are doped in the WSe_2_ channel. To check these conjectures, *V*_G0_ and *V*_G1_ were independently varied from −10 to 10 V in steps of 1 V, for a total of 441 configurations. At each configuration, *V*_D1_ was swept from −2 to 2 V in steps of 0.04 V, and the current *I*_D1_ was recorded. The source electrode was grounded. The test results are shown as a 4-dimensional data cube in Figure 2a, in which the color represents the absolute value of *I*_D1_. Figure S6a shows the back view of the data cube.

First, we check the symmetric configurations of gate voltages by chopping through the data cube to expose the diagonal plane of *V*_G0_ = *V*_G1_ (Figure 2b), which confirm that both hole and electron doping can make the WSe_2_ channel conductive (ambipolar FET) irrespective of the polarities of *V*_D1_ when the gate voltages exceed certain thresholds (yellow dots, see the caption of Figure 2). The extracted transfer characteristic curve from Figure 2b at a fixed *V*_D1_ of 1.04 V shows that the on/off ratio is >10^3^ for p-channel (holes accumulated) and >10^4^ for n-channel (electrons accumulated) (see Figure S6b), which indicates this ETH device has a high quality. We also notice that the leakage currents of *I*_G0_ and *I*_G1_ are always below ∼10^–10^ A level in all tests (Figure S6c). Then, we check the asymmetric scenario. Figure 2d is the front plane of the data cube, where *V*_G0_ is fixed at 10 V. It is observed that when *V*_G1_ is positive and exceeds certain thresholds, the WSe_2_ channel can be turned on regardless of the polarities of *V*_D1_. It should be noted that in this case *V*_G0_ and *V*_G1_ have the same polarity but different amplitudes that are distinct from the symmetric configuration where *V*_G0_ = *V*_G1_. Nevertheless, when *V*_G1_ is lower than −5 V, only positive *V*_D1_ up to a threshold can turn the WSe_2_ channel on. The counterpart of fixing *V*_G0_ at −10 V also shows similar results apart from a negative *V*_D1_, making the WSe_2_ channel conductive (Figure 2e).

**Figure 3:**
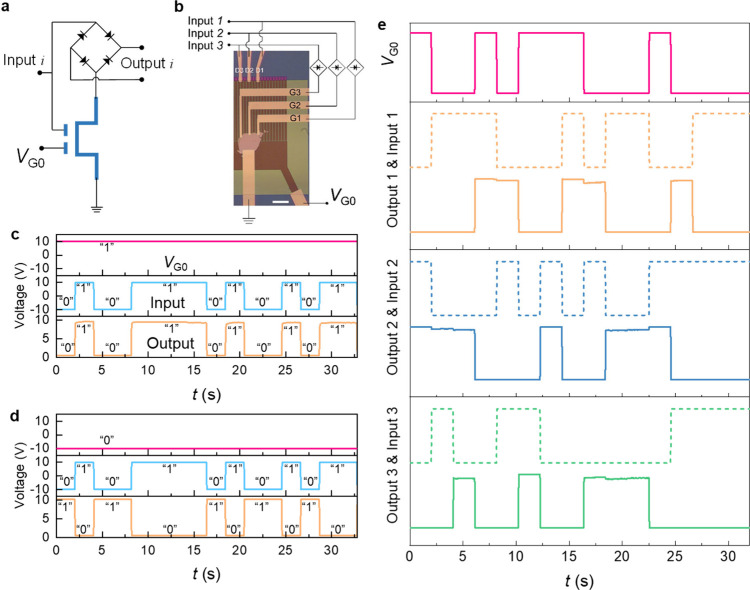
Implementing logic operations and encryption using ETH devices. (a) A circuit diagram. (b) A circuit integrated with a 3-input ETH device for implementing encryption. Scale bar: 4 μm. (c) Demonstration of plain code by setting *V*_G0_ = 10 V as the secret key. (d) To invert the input by setting *V*_G0_ = −10 V as the secret key. (e) Applying a random voltage sequence for *V*_G0_ as the secret key to encrypt 3 inputs in parallel. Dashed lines and solid lines indicate the inputs and outputs, respectively.

We further highlight this observation by extracting the four vertical edges of the data cube (Figure 2c and linear scale plot in Figure S6d), in which the magnitudes of *V*_G0_ and *V*_G1_ are maintained at 10 V. When 10 and −10 V are applied to *V*_G0_ and *V*_G1_ (red curve), respectively, a rectification effect is observed, i.e., a diode. When *V*_G0_ and *V*_G1_ are swapped, the direction of rectification is also swapped (blue curve). In contrast, no rectification appears for the same polarity applied to G0 and G1 (black and green curves). The rectification effects are ascribed to the formation of a p–n junction when *V*_G0_ and *V*_G1_ are set with different polarities. For instance, the red curve is related to Figure 1e,h, where the drain electrode contacts the top WSe_2_ segment, where holes are accumulated (p-channel); the WSe_2_ bottom segment is the location where electrons are accumulated (n-channel), and thus, by applying a positive *V*_D1_, the diode would be forward biased. The same analysis is also suitable for the blue curve. The rectification data are fitted to the Shockley diode equation,^44^ giving an ideality factor of *n* ∼ 2 for both n–p and p–n *I*_D1_–*V*_D1_ curves, indicating that *I*_D1_ is mostly limited by recombination rather than diffusion (Supplementary Note 1). In Figure S7, we reproduce the electrical results based on device 3, a 4-gate device with a 9-layer (∼6.4 nm) WSe_2_ flake (Figure 3b). We note that the electrically tunable rectification effects are still observed when the split distance of the gates even increases to 2.4 μm although the on-state currents are lower compared with the cases with shorter split distances (Figure S8).

In MOSFETs, only the gate voltage switches the channel on/off. By contrast, both electrical measurements and KPFM results unambiguously demonstrated that the drain voltage can also determine the conductive status of the WSe_2_ channel, which makes the ETH device a unique logic unit at the component level. Next, we extract the logic rules in our ETH devices. The custom in conventional electronics is obeyed, taking positive voltage as the logic state “1” and negative voltage as the logic state “0”; thereby, e.g., Figure 2d is given with fixing *V*_G0_ at “1”. In the first and third quadrants of Figure 2d, *V*_D1_ and *V*_G1_ have the same polarities, resulting in the same logic states. The two areas outlined by the white dashed curves indicate that *V*_D1_ = *V*_G1_ = “1” can switch the WSe_2_ channel on, whereas *V*_D1_ = *V*_G1_ = “0” turns off the channel. Likewise, Figure 2e with fixed *V*_G0_ = “0” shows the opposite results. Figure 2f concludes the rules by further taking channel “on” as logic “1” and “off” as logic “0”, which is a truth table of the XNOR logic gate and reveals that the information input by “*V*_D1_ = *V*_G1_” can be dynamically converted by switching the logic states of *V*_G0_.

Note that the KPFM results and electrical measurements are extracted from a single junction in a 2-gate ETH device, but the logic principle is also applicable for every junction of the device having multiple gates, such as the devices in Figures 1b and 3b, where G0 is the global gate used to form each p–n junction in combination with each local gate. The parallel distribution can effectively avoid crosstalk between the local gates (Supplementary Note 2). When the voltage on the global gate is altered, all of the p–n junctions are affected. This attribute gives the multiple-gate ETH devices the capacity for parallel logic operation, by contrast, which would otherwise require complicated cascades of MOSFETs or 2-gate ETH devices (Supplementary Note 3).^45^

## Parallel Logic Operation and Information Encryption

To perform the logic operations indicated by Figure 2f, we designed a compact circuit integrated with our ETH devices; see a circuit diagram in Figure 3a. It is found in Figure 2d,e that *V*_D1_ and *V*_G1_ have the same polarity, so two terminals of a rectifier connect a local gate and a drain to realize “*V*_D1_ = *V*_G1_” and serve as an input port. *V*_G0_ serves as the other input port. Rectifiers also play the role of resistive dividers, which can convert the WSe_2_ channel on/off states to partial voltages being accessed via the other two terminals as the output. Meanwhile, the rectification effect of rectifiers always makes the output voltages positive. It is important to note that the extra supply voltage (*V*_CC_) is avoided in our circuit design, resulting in no static power consumption.

Next, we experimentally demonstrate that our ETH device can achieve the functions shown in Figure 2f. See Figure 3c,d, the experiment results from device 1 with 2 gates, in which the logic operations manifested by Figure 2f are reproduced well. For example, in Figure 3d, we set *V*_G0_ = “0” (pink line), and the circuit outputs “1” and “0” (brown curve) if “0” and “1” are input into the port of “*V*_D1_ = *V*_G1_” (blue curve), respectively. We further consider these logic operations as information encryption processes, as follows.

In Figure 3c, the input was “01001111 01001011” representing the ASCII code of “OK”, and then a plain code was output upon fixing *V*_G0_ = “1”. Plain code refers to the output having an identical form as the input despite the output voltage range are different. Note that plain code is widely used in public radio and HTTP. By contrast, in Figure 3d, by fixing *V*_G0_ = “0”, the input was processed using an invert operation to output “10110000 10110100”. Intrinsically, setting *V*_G0_ = “0” or “1” is to configure a secret key, even if the secret keys in Figures 3c and 3d are too simple to protect the encrypted information. In addition, the 16-bit data representing “OK” has to be serially processed one by one because device 1 has only one input port (*V*_G0_ is used for the secret key), resulting in limited efficiency. A better alternative is to use fluctuating sequences as the secret keys in a multiple-input ETH device.

In Figure 3b, a circuit with a three-input ETH device (device 3 with 4 gates) is established based on the basic circuit shown in Figure 3a. Since device 3 has 3 input ports, 3 bits can be processed at the same time. Moreover, G0 is a global gate; therefore, the secret key loaded on it can be used to encrypt all inputs. In Figure 3e, a random sequence (pink curve) generated by a stochastic algorithm is loaded onto the global G0 terminal to process 3 input data flows in parallel (dashed lines). This secret key applies to randomly selected input bits invert or plain operations, which output unreadable ciphertexts (solid lines). It can be found that every input can independently exert logic operations with the input from the G0 terminal, showcasing no noteworthy crosstalk among the inputs. Thus, in principle, adding more input terminals is feasible to enhance the encryption capacity of ETH devices. For example, Figure 1b shows a 9-input ETH device and a byte can be encrypted simultaneously (note that we did not test this device because of the limited capacity of our measurement system). It should be emphasized that the logic operations (plain and invert) in our ETH devices are simple but nevertheless do not mean encryptions are not secure because the determining factor of security is the algorithms used to generate the secret keys.^46^

**Figure 4:**
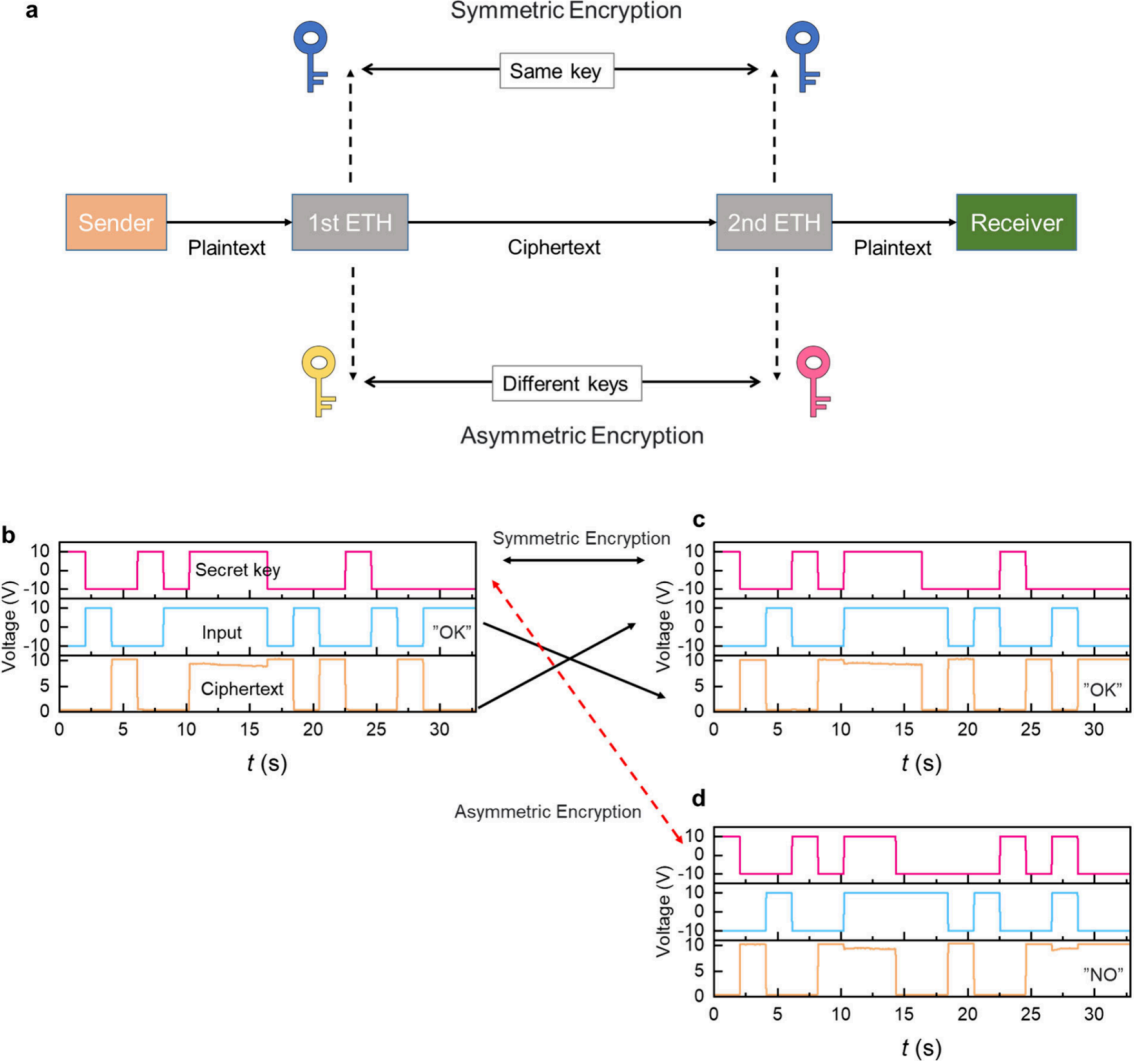
Confidential communication based on two ETH devices. (a) A schematic diagram of confidential communication as well as illustrating symmetric encryption and asymmetric encryption in this data transmission route integrated with two ETH devices. (b–d) Experimental results of confidential communication. (b) is associated with the first ETH device. (c) and (d) are associated with the second ETH device when it works on symmetric encryption and asymmetric encryption, respectively.

On the contrary, thanks to the simple logic operations, the ETH devices can further become a decryptor. Figure 4a shows a confidential communication route integrated with two ETH devices. The ciphertext generated by the first ETH device can be translated by the second ETH device. We applied two different encryption strategies in this route, i.e., symmetric and asymmetric encryptions, which are widely used in daily life. Specifically, by configuring the same (different) secret key in the second ETH device as that in the first ETH device, symmetric (asymmetric) encryption can be established. Figure 4b,c experimentally demonstrates symmetric encryption. Supposing the sender wanted to transmit “OK” to the receiver (Figure 4b), a random voltage sequence (pink curve) was configured on G0 of the first ETH device as the secret key, and the ASCII code representing “OK” (blue curve) was input. Then, the ciphertext (brown curve) would be generated and transmitted. Even if the ciphertext was eavesdropped during the transmission, the cheater cannot get the right information without the right secret key. Subsequently, in Figure 4c, the ciphertext was sent into the second ETH device as the inputs before arriving at the receiver, and then by configuring the same secret key as in the first ETH device, the ciphertext was translated as “OK”. Thus, a confidential communication was completed. Analogously, in Figure 4d, a different secret key was used in the second ETH device (asymmetric encryption), showing more diversified results. Note that our ETH devices are physical. Compared with software encryptors, physical ones are more secure and efficient.

## Supporting Information Available

Experimental section, additional KPFM results and electrical transport data, and supplementary notes (PDF)
