# Supplementary material for: Parallel Logic Operations in Electrically Tunable Two-Dimensional Homojunctions
Source: Nano Lett. 2024 Oct 30;24(45):14420–6. doi: 10.1021/acs.nanolett.4c04337 (PMC11565736; doi:10.1021/acs.nanolett.4c04337)
Supplement: Supplementary file 1 — nl4c04337_si_001.pdf [file nl4c04337_si_001.pdf]

Supplementary Information

Parallel logic operations in electrically tunable two-dimensional homojunctions

Yuliang Chen[1*], Zhong Wang[1], Chongwen Zou[2], Stuart S. P. Parkin[1*]

[1]Max Planck Institute of Microstructure Physics 06120, Halle, Germany

[2]National Synchrotron Radiation Laboratory, School of Nuclear Science and Technology, University of Science and Technology of China 230029, Hefei, China

[*]Corresponding e-mail: yuliang.chen@mpi-halle.mpg.de, stuart.parkin@mpi-halle.mpg.de

Contents

Experimental Section

Fig. S1-9

Supplementary Notes 1-3

Experimental Section

Device fabrication. The WSe_2 crystal was grown using a custom chalcogen flux method[1-2]. Standard electron-beam lithography (EBL, Raith Pioneer 2) was used to define the split gates, followed by a Ti(1 nm)/Au(9 nm) deposition (Scia Coat 200) on a Si/SiO_2 (300 nm) substrate. The split gap of the prepared gating electrodes was measured by SEM (Raith Pioneer 2). The same procedures were used to deposit a 70 nm Al_2O_3 dielectric layer on top of the prepared gates (Scia Coat 200). The few-layered WSe_2 flakes were mechanically exfoliated on Si/SiO_2 (285 nm) substrates. The thickness of the flakes was initially identified by optical contrast compared with a standard sample (Supplementary Fig. 9), followed by Raman (Horiba Labram HR Evolution), photoluminescence (PL, Horiba Labram HR Evolution), and atomic force microscopy (AFM, Oxford Cypher ES) measurements[3]. Then, the exfoliated WSe_2 flakes were transferred to the top of the Al_2O_3 dielectric layer by a dry transfer technique[4]. Finally, a 3rd EBL procedure was employed to define the drains and source, followed by Ti(1 nm)/Au(70 nm) deposition.

Characterization. KPFM measurements were carried out using an Oxford Cypher ES. The drain and source electrodes were shorted and grounded during the tests. The gating voltages were independently applied on the gates by two Keithley 2400 sourcemeters. The measurements of electrical properties and encryptions were conducted by a homemade system integrated with multiple sourcemeters controlled by the compiled Labview files. A 10s waiting time was set in the Labview files after changing the configuration of gating voltages to ensure the system was stable. All characterizations were conducted at room temperature.

Supplementary Figures

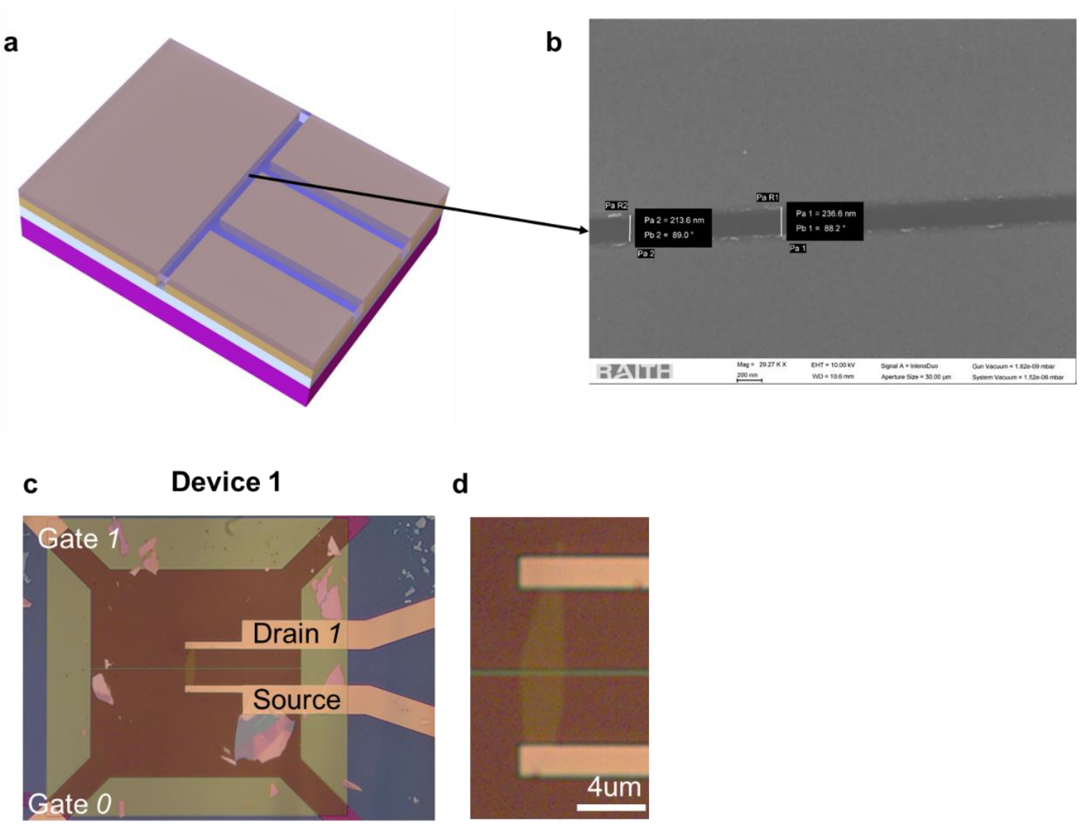

Figure S1. a, Schematic illustration of the arrangement of gates in the multiple-gate ETH devices. **b**, The gap between the two gates is ~230 nm as determined by SEM. **c**-**d**, A 2-gate ETH device (Device 1) and details.

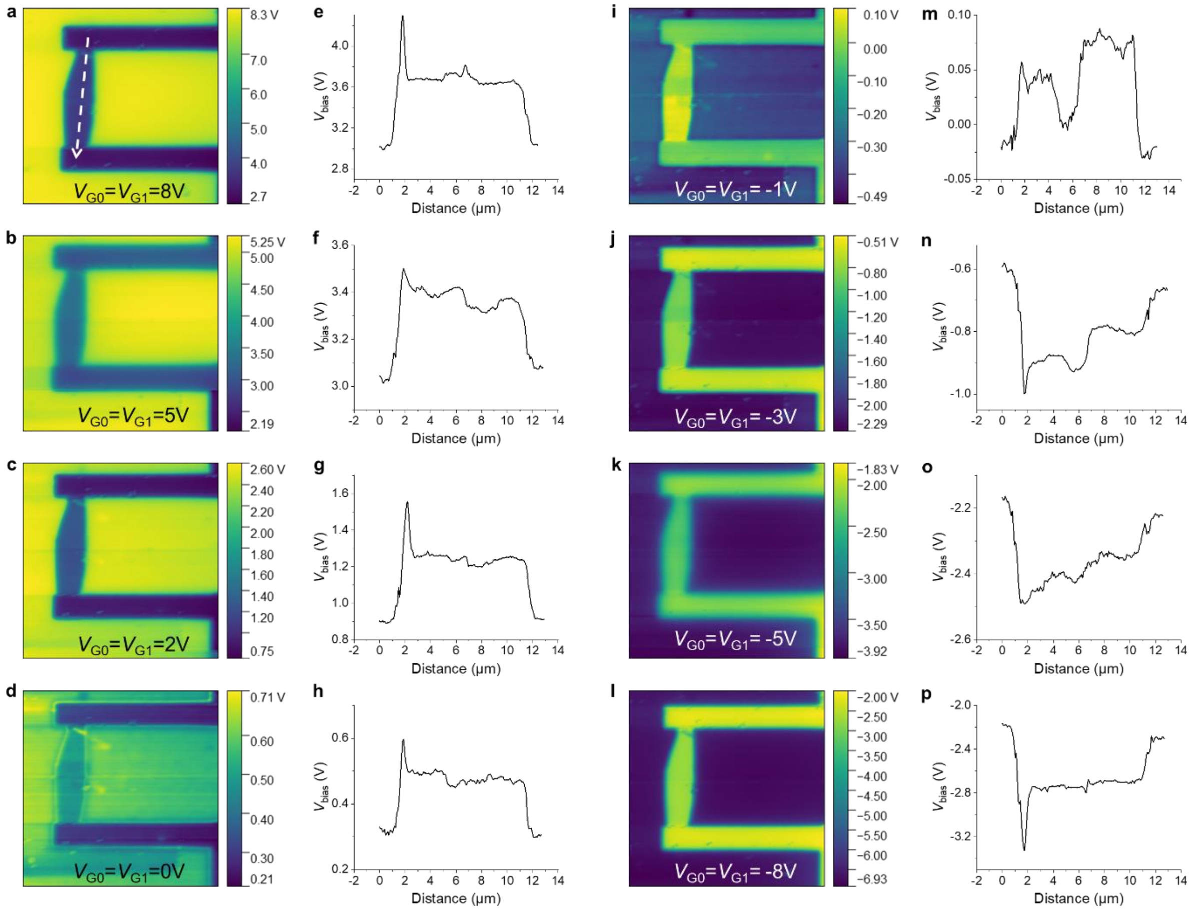

Figure S2. a-**d** and **i**-**l**, Additional KPFM scanning results from Device 1. **e**-**h** and **m**-**p**, The corresponding extracted data from **a**-**d** and **i**-**l** along the line indicated by the dashed arrow in **a**. The head and tail of the arrow are on the Au electrodes, and we used V_{bias} of the Au electrodes as a reference for processing the experimental data.

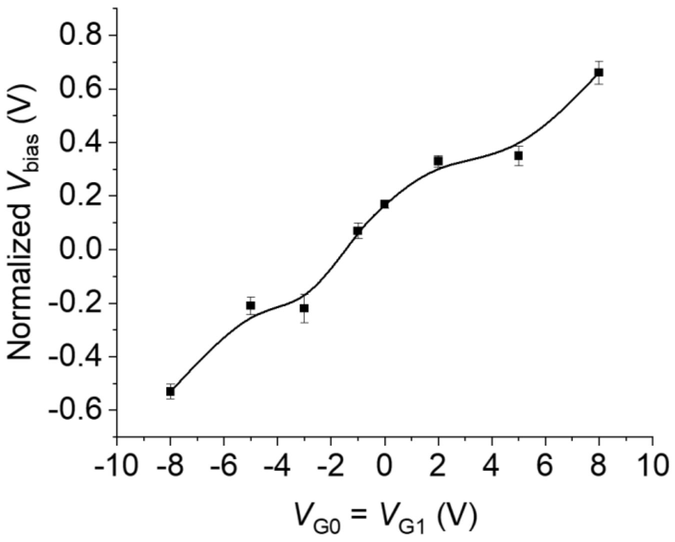

Figure S3. Evolution of surface potential of WSe_2 against gating voltages by taking V_{bias} of Au as the reference to normalize the V_{bias} of Fig. S2. The solid line guides the eyes. The error bars represent the standard deviation.

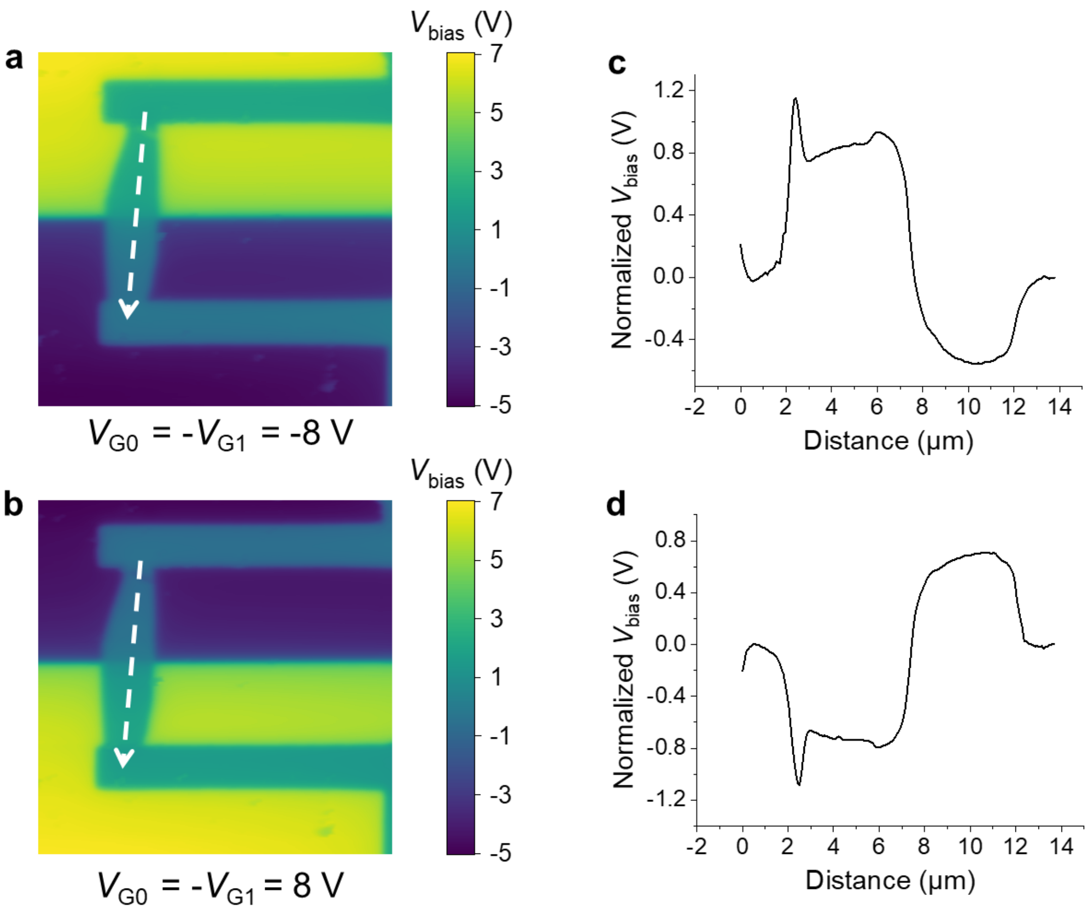

Figure S4. **a**-**b**, KPFM results of Device 1 by applying opposite voltages on the two gating electrodes. **c**-**d**, Data indicated by dashed arrows in **a** and **b** are replotted in **c** and **d**, respectively.

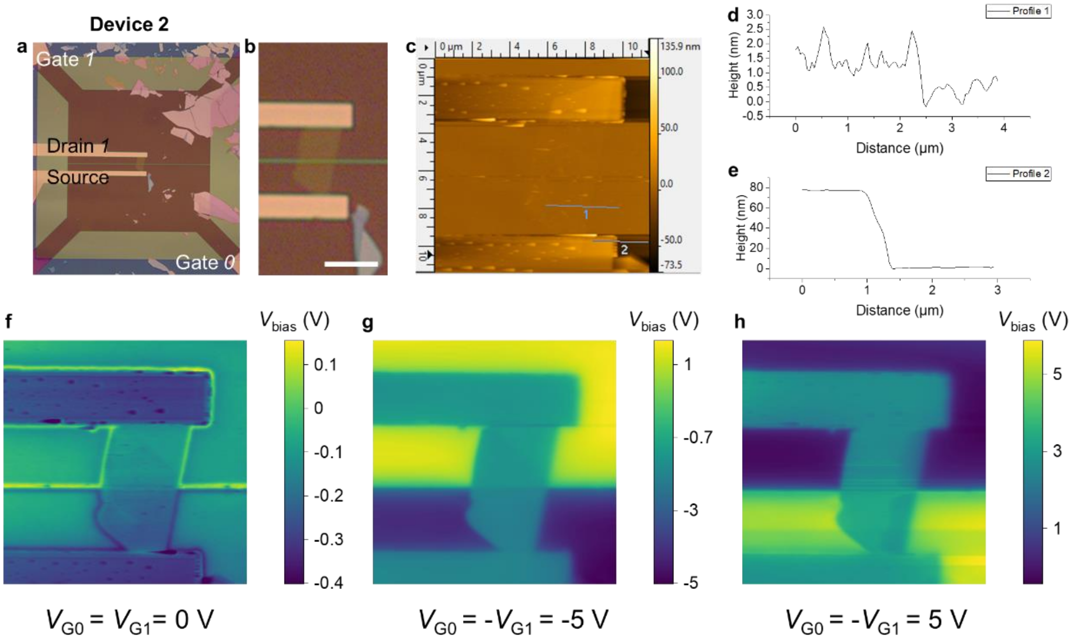

Figure S5. a-b, Additional 2-gate ETH device (Device 2) with a bilayer WSe_2 flake as the channel. Scale bar, 4 μm. **c**, Morphology of Device 2 as tested by AFM. **d-e**, Corresponding profiles in **c**, confirming the ~1.4 nm thickness for bilayer and indicating a ~70 nm thickness for the electrodes. **f-h**, KPFM results of Device 2.

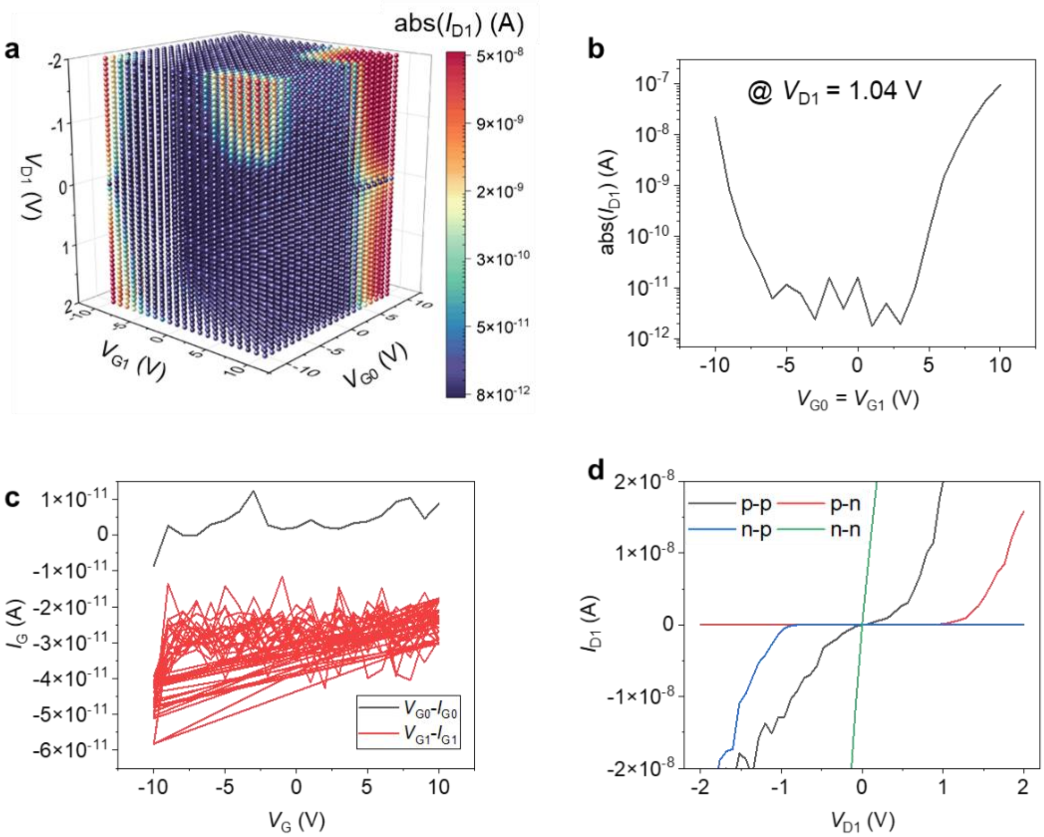

Figure S6. a, Back view of the data cube in Fig. 2a of the main text. **b**, In Fig. 2b of the main text, further extracting the data at V_{D1} = 1.04 V. **c**, The leakage currents from gating terminals while measuring the data cube of **a**. We compiled a Labview file to automatically conduct electrical experiments. In that Labview file, we set V_{G0} as the most outer loop sweeping from -10 V to 10 V, and set V_{G1} as the second outer loop sweeping from -10 V to 10 V; at each configuration, sweeping V_{D1} and measuring I_{D1} in the inner loop. This is the reason why I_{G1} has more data than I_{G0}. **d**, Plotting V_{D1}-I_{D1} in linear coordinate corresponding to 4 vertical edges of **a**.

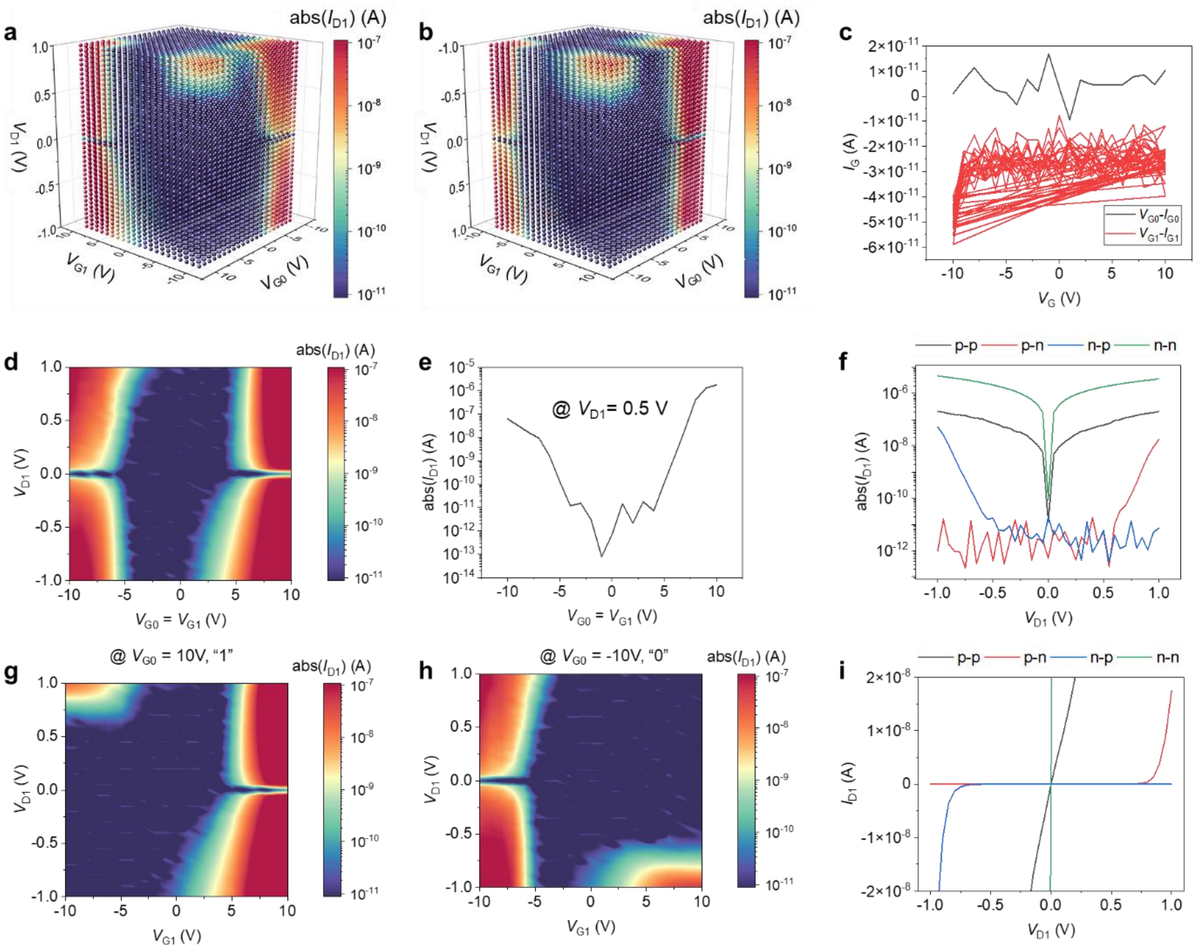

Figure S7. A set of electric measurements for Device 3 with 4 gates.

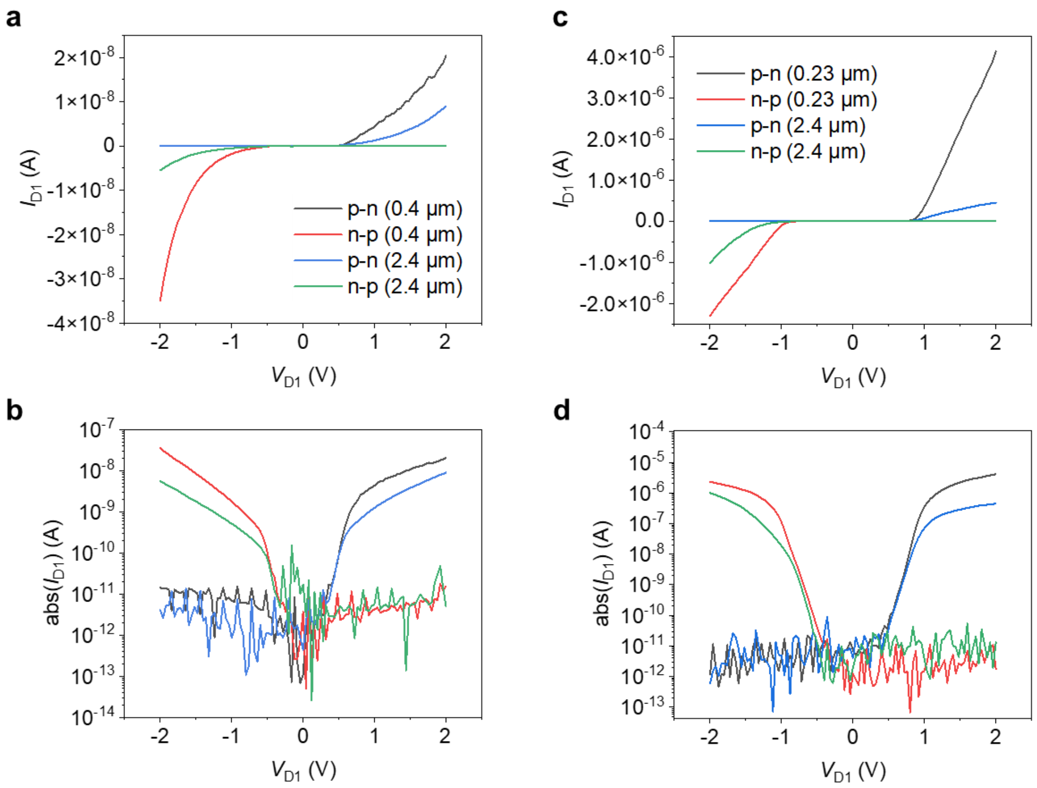

Figure S8. *I*-*V* curves for two devices (Device 4 and Device 5). **a** and **b** are for Device 4 with a WSe_2 trilayer (~2 nm) covering multiple gates with different split distances, indicated in the brackets. In analogy to **a** and **b**, **c** and **d** are for Device 5 with a 9-layered WSe_2 sheet.

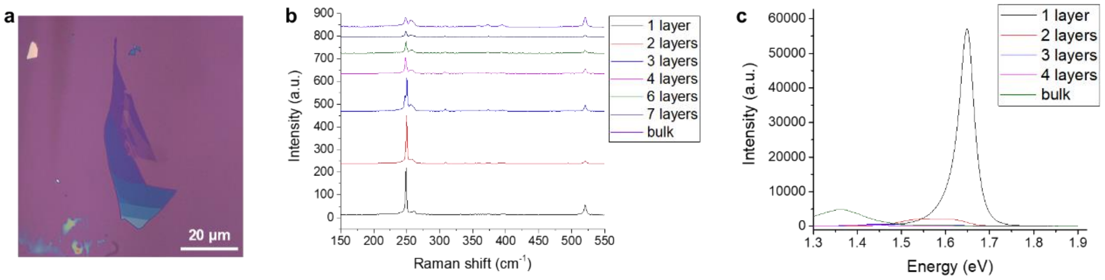

Figure S9. a, Optical image of a terraced WSe_2 flake as the standard sample for prechecking the layer of exfoliated flakes. **b**, Raman and **c,** PL results evolve with the number of layers.

Supplementary Note 1

I-V characteristic of WSe_2 p-n junction

The I-V characteristics of the p-n junctions in Fig. S6d can be fit to the Shockley diode equation[5],

$$I(V) = I_S(e^{\frac{V}{nV_T}} - 1),$$

where V is the applied bias, I_S is the reverse saturation current, V_T is the thermal voltage (≈ 26 mV at room temperature), and n is the diode ideality factor. The experimental output curves should include a series resistor R_S, which consists of the electrode/WSe_2 contacts and the p- and n- channels themselves. An explicit equation is,

$$I(V) = \frac{nV_T}{R_S} W_0 \left(\frac{I_S R_S}{nV_T} e^{\frac{V + I_S R_S}{nV_T}} \right) - I_S$$

$W_0()$ is the 0th branch of Lambert W-function. The fitting curves are drawn in solid curves in Fig. S10. $n \approx 2$ indicates the current is mostly limited by recombination instead of diffusion[6].

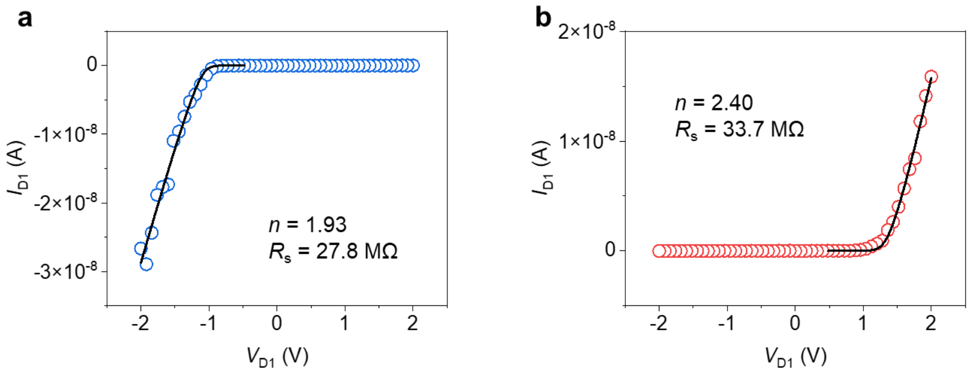

Figure S10. Fitting results of I-V characteristics of the WSe_2 p-n junctions. The empty circles are the measured data and the solid lines are the fitted curves.

Supplementary Note 2

Parallel encryption in our ETH devices

To realize parallel encryption in our ETH devices, the crosstalk among inputs has to be avoided, for which we can increase the split gap or etch the WSe_2 segments between every two local gates. However, another way, keeping the same split as the one between global and local gates (~230 nm for our actual devices), is still available. The crosstalk is avoided in the ETH devices because of the formed p-n junctions of every two local gates.

See Fig. S11b, setting V_{G0} = "0", V_{G1} = "1", and V_{G2} = "1", because D1 and D2 have the same positive voltages as G1 and G2, respectively, the two p-n junctions produced by G0-G1 and G0-G2 are working under reversed condition (Fig. S11b). Under this situation, two outputs are the same, even if there is a crosstalk between input-1 and input-2, which would not impact the outputs.

Then, in Fig. S11c, the p-n junction between G0 and G1 is maintained. What will happen if setting V_{G2} to "0"? The p-n junction between G0 and G2 is erased, accordingly, the channel between G0 and G2 is switched on. Also, a new p-n junction is formed between G1 and G2. However, it is under reverse bias resulting in suppressed crosstalk between inputs. The same analysis can be done for other situations and more inputs, so the crosstalk among inputs is negligible, which is experimentally validated, as shown in Fig. 3e of the main text.

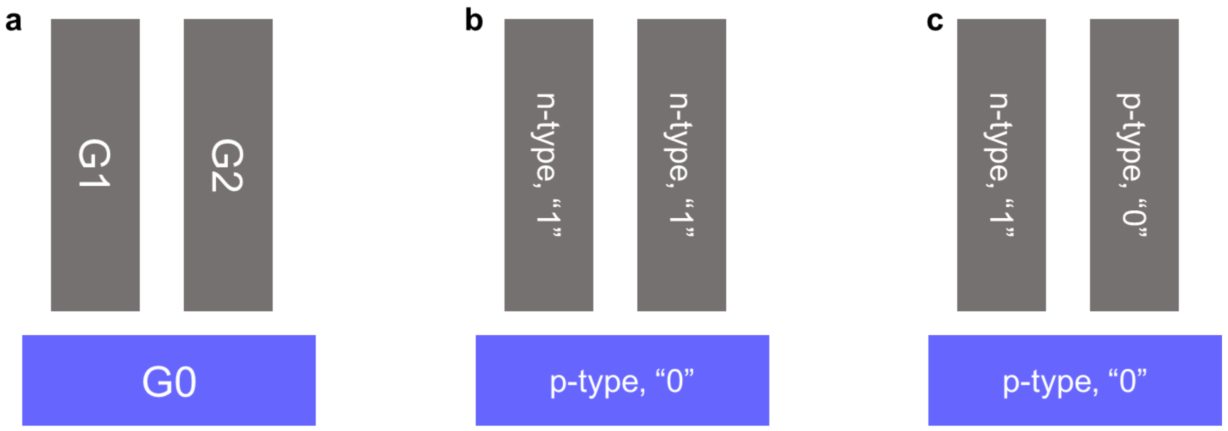

Figure S11. Schematic illustration for noting our ETH devices can do the logic operations in parallel for multiple inputs without crosstalk.

Supplementary Note 3

Ultra-high integration level in ETH devices

Fig. 2f in the main text is a truth table of the XNOR logic gate. Therefore, the functions of ETH devices can be realized by cascading MOSFETs, in principle. 4 transistors at least are needed to realize a XNOR logic gate (Fig. S12)[7], which is capable of our 1-input ETH device. We can cascade 3 such XNOR logic gates to work as our 3-input ETH device (Device 3), that is, 12 transistors are needed. For the 9-input ETH device in Fig. 1b of the main text, 36 transistors are needed. Our ETH devices have an ultra-high integration level and are free from complicated cascading designs.

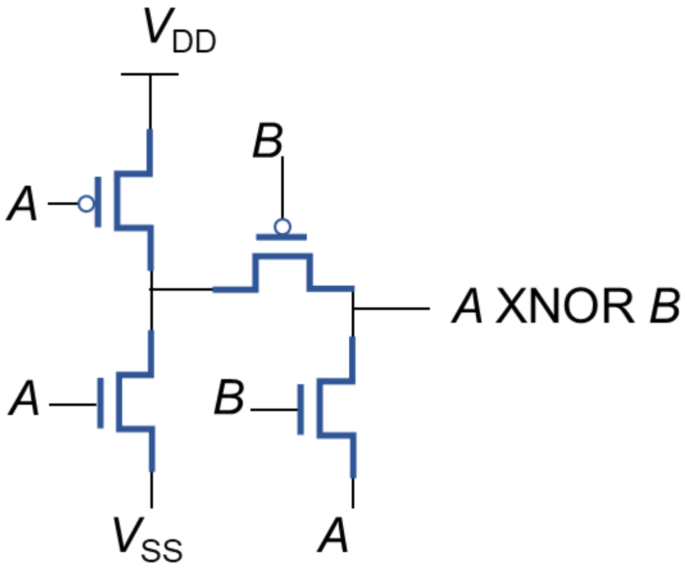

Figure S12. 4-transistor XNOR circuits by cascading conventional MOSFETs.

References

1. Edelberg, D.; Rhodes, D.; Kerelsky, A.; Kim, B.; Wang, J.; Zangiabadi, A.; Kim, C.; Abhinandan, A.; Ardelean, J.; Scully, M.; Scullion, D.; Embon, L.; Zu, R.; Santos, E. J. G.; Balicas, L.; Marianetti, C.; Barmak, K.; Zhu, X.; Hone, J.; Pasupathy, A. N., Approaching the Intrinsic Limit in Transition Metal Diselenides via Point Defect Control. *Nano letters* **2019,** *19* (7), 4371-4379.
2. Gustafsson, M. V.; Yankowitz, M.; Forsythe, C.; Rhodes, D.; Watanabe, K.; Taniguchi, T.; Hone, J.; Zhu, X.; Dean, C. R., Ambipolar Landau levels and strong band-selective carrier interactions in monolayer WSe2. *Nature materials* **2018,** *17* (5), 411-415.

3. Tonndorf, P.; Schmidt, R.; Bottger, P.; Zhang, X.; Borner, J.; Liebig, A.; Albrecht, M.; Kloc, C.; Gordan, O.; Zahn, D. R.; Michaelis de Vasconcellos, S.; Bratschitsch, R., Photoluminescence emission and Raman response of monolayer MoS(2), MoSe(2), and WSe(2). *Opt Express* **2013,** *21* (4), 4908-16.
4. Zomer, P. J.; Guimarães, M. H. D.; Brant, J. C.; Tombros, N.; van Wees, B. J., Fast pick up technique for high quality heterostructures of bilayer graphene and hexagonal boron nitride. *Applied Physics Letters* **2014,** *105* (1), 013101.
5. Banwell, T. C.; Jayakumar, A., Exact analytical solution for current flow through diode with series resistance. *Electronics Letters* **2000,** *36* (4), 291.
6. Enke, L.; Bingsheng, Z.; Jinsheng, L., *The physics of semiconductors*. Publishing House of Electronics Industry: Beijing, 2003.
7. Lin, J.-F.; Hwang, Y.-T.; Sheu, M.-H.; Ho, C.-C., A Novel High-Speed and Energy Efficient 10-Transistor Full Adder Design. *IEEE Transactions on Circuits and Systems I: Regular Papers* **2007,** *54* (5), 1050-1059.
